# Population Pharmacokinetic Analysis of Yimitasvir in Chinese Healthy Volunteers and Patients With Chronic Hepatitis C Virus Infection

**DOI:** 10.3389/fphar.2020.617122

**Published:** 2021-01-28

**Authors:** Xiao-duo Guan, Xian-ge Tang, Ying-jun Zhang, Hong-ming Xie, Lin Luo, Dan Wu, Rui Chen, Pei Hu

**Affiliations:** ^1^ Clinical Pharmacology Research Center, Peking Union Medical College Hospital, Peking Union Medical College and Chinese Academy of Medical Sciences, Beijing, China; ^2^ Beijing Key Laboratory of Clinical PK and PD Investigation for Innovative Drugs, Beijing, China; ^3^ State Key Laboratory of Anti-Infective Drug Development, Dongguan, China; ^4^ Sunshine Lake Pharma Co., Ltd., Dongguan, China

**Keywords:** population pharmacokinetic, yimitasvir, hepatitis C virus, non-structural protein 5A inhibitor, phoenix NLME

## Abstract

Yimitasvir is a novel, oral hepatitis C virus (HCV) non-structural protein 5A inhibitor for the treatment of chronic HCV genotype 1 infection. The objective of this analysis was to develop a population pharmacokinetic model of yimitasvir in Chinese healthy volunteers and HCV infection patients. The model was performed using data from 219 subjects across six studies. Nonlinear mixed effects models were developed using Phoenix NLME software. The covariates were evaluated using a stepwise forward inclusion (*p* < 0.01) and then a backward exclusion procedure (*p* < 0.001). A two-compartment model with sequential zero-first order absorption and first-order elimination reasonably described yimitasvir pharmacokinetics (PK). The apparent oral clearance and central volume of distribution were 13.8 l·h^−1^ and 188 l, respectively. The bioavailability (F) of yimitasvir decreased 12.9% for each 100 mg dose increase. Food was found to affect absorption rate (Ka) and F. High-fat meal decreased Ka and F by 90.9% and 38.5%, respectively. Gender and alanine aminotransferase were identified as significant covariates on apparent oral clearance. Female subjects had lower clearance than male subjects. Zero-order absorption duration was longer in healthy volunteers (2.17 h) than that in patients (1.43 h). The population pharmacokinetic model described yimitasvir PK profile well. Food decreased Ka and F significantly, so it was recommended to take yimitasvir at least 2 h before or after a meal. Other significant covariates were not clinically important.

## Introduction

Chronic infection with hepatitis C virus (HCV) is a public health concern in the world, which can lead to liver cirrhosis, and/or hepatocellular carcinoma (primary liver cancer) (Jafri and Gordan, 2018). In 2002, National Institutes of Health Consensus Development Conference Statement reported more than 184 million persons had HCV infection (National Institutes of Health Consensus Development Conference Statement: management of hepatitis C 2002). An epidemiology in 2015 estimated that 1.0% of the world population corresponding to approximate 71 million people were active cases (Polaris Observatory HCV Collaborators, 2017). Every year three to four million people are newly infected and approximately 350,000 deaths occur (Mohd et al., 2013). HCV demonstrates great genetic diversity with seven genotypes and at least 67 subtypes (Jafri and Gordan, 2018). Overall, genotype 1 dominates with 44% of infections, followed by genotype 3 (25%) and 4 (15%) (Polaris Observatory HCV Collaborators, 2017). In China, it is estimated that at least 25 million individuals infected with HCV (Bian et al., 2017) and genotype 1b is the most common type (56.8%), followed by genotype 2 (24.1%) and 3 (9.1%) (Chinese Society of Hepatology and Chinese Society of Infectious Diseases, 2015).

**FIGURE 1:**
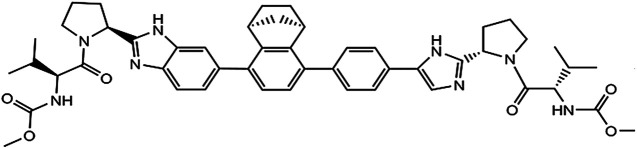
The chemical structure of yimitasvir.

Yimitasvir is a novel, oral HCV non-structural protein 5A (NS5A) inhibitor for the treatment of chronic HCV genotype 1 infection in combination with sofosbuvir. The chemical structure of yimitasvir is shown in Figure 1. The pharmacokinetic profile of yimitasvir has been evaluated in healthy volunteers and patients with chronic HCV infection (Zhang et al., 2018; Zhao et al., 2019). Following fasted single oral dose of yimitasvir in healthy volunteers, yimitasvir was absorbed with a peak concentration (C_max_) 3.5–4.0 h post-dose. Area under the concentration-time curve (AUC) and C_max_ increased in a dose-proportional manner from 30 to 100 mg but a less than proportional manner from 100 to 600 mg (single ascending dose [SAD] study) (Zhao et al., 2019). Similarly, less than dose-proportional manner was found in multiple ascending dose (MAD) study in the range of 100–400 mg once daily for seven consecutive days. However, the result from phase 1b study in patient population showed that yimitasvir exhibited near dose-proportional increase in exposure from 30 to 200 mg administered during the night (4 h after dinner) (Zhang et al., 2018). Yimitasvir was approximately 79.2–86.6% bound to human plasma proteins and the binding was independent of drug concentration over the range of 100–2000 ng ml^−1^. No metabolism of yimitasvir was detected *in vitro* during incubations with hepatic microsomes from mice, rats, dogs, monkeys and humans. Less than 0.04% of yimitasvir was recovered in urine as the parent drug through 7 days post-dose and fecal excretion of parent drug was the major route of elimination (Zhao et al., 2019). The terminal half-life (t_1/2_) of yimitasvir was 13.4–19.7 h, supporting once daily dosing schedule. Steady state was achieved by day 5 following the once daily dosing regimen. The accumulation ratio was 1.32–1.34, consistent with half-life. A high-fat meal reduced absorption rate with T_max_ occurring at 5–12 h post-dose and resulted in approximate 50% and 63% decrease in yimitasvir AUC and C_max_, respectively (Zhao et al., 2019). Yimitasvir is a substrate and inhibitor of the drug transporter P-glycoprotein (P-gp). Yimitasvir is a weak inhibitor of cytochrome P450 (CYP) 2C8, but does not inhibit CYPs 1A2, 2B6, 2C9, 2C19, 2D6 and 3A4. Yimitasvir may be a weak inducer of CYP3A4.

In phase 2 study, yimitasvir 100 or 200 mg was administered once daily for 12 weeks in combination with 400 mg sofosbuvir in patients with chronic HCV infection. Similar to other HCV NS5A inhibitors such as velpatasvir (Brieva et al., 2017) and ledipasvir (German et al., 2016), yimitasvir PK profile was not affected by co-medication of sofosbuvir. The primary endpoint of phase 2 study was sustained virologic response [HCV RNA less than lower limit of quantification (LLOQ)] 12 (SVR12) weeks after the completion of treatment. SVR12 rates were achieved 100% in both 100 mg yimitasvir/400 mg sofobuvir and 200 mg yimitasvir/400 mg sofobuvir groups. The adverse reaction rates were comparable between 100 mg (35.9%) and 200 mg (36.9%) groups. The most common adverse reactions were neutropenia (3.9%), leukopenia (3.1%), hypercholesterolemia (3.1%) and fatigue (3.1%). All of these adverse reactions were grade one or two in severity. In summary, no dose-response relationship for efficacy and safety was observed in phase 2 study.

The aim of our study was to develop a population PK model to characterize yimitasvir PK in Chinese population and to identify the significant covariates affecting yimitasvir PK. This model will be further updated with much more patient PK data from phase 3 study and be used for predicting individual subject exposure for efficacy and safety exposure-response analysis of yimitasvir.

## Methods

### Patients

The current population PK analysis of yimitasvir was performed using rich and sparse PK samples collected from six clinical pharmacology trials [4 phase 1 (Zhao et al., 2019), 1 phase 1b (Zhang et al., 2018) and 1 phase 2 studies] in Chinese healthy volunteers and HCV-infected patients. Rich sampling entailed serial blood sampling at defined time points, and sparse sampling (single sample) only in phase 2 study entailed blood collection at every study visits. Patients with genotype 1 HCV infection were eligible, while patients with prior use of direct-acting antiviral agents (DAA) for HCV infection treatment were excluded in phase 1b and phase 2 studies. Detailed study design and sampling schedule are shown in Table 1.

**TABLE 1:** Participants, dosing regimens and pharmacokinetic sampling plans for studies included in the population pharmacokinetic analysis.

Study code	Subjects	Study design	Dosing regimen	No. of subjects^a^	Food status	PK sampling time	No. of PK samples
Phase 1							
CTR20140854 (NCT03462173)	Healthy volunteers	R, DB, PC, SAD	SD, 6A + 2P	n = 24	Fasted at least 10 h	Pre-dose and post-dose 0.5, 1, 2, 3, 4, 5, 6, 8, 10, 12, 24, 36, 48, 72, 96, 120 and 144 h	Rich: 432
			30, 100, 200, 400 mg				
CTR20150048	Healthy volunteers	R, DB, PC, MAD	QD × 7 days, 9A + 3P	n = 18	Fasted at least 10 h	Day 1 pre-dose and post-dose 0.5, 1, 2, 3, 4, 5, 6, 8, 10, 12 and 24 h; day 5, 6, 7 pre-dose; day 7 post-dose 0.5, 1, 2, 3, 4, 5, 6, 8, 10, 12, 24, 36, 48, 72, 96 and 120 h	Rich: 558
			100, 200 mg				
CTR20170932	Healthy volunteers	R, DB, PC	SD, 6A + 2P 600 mg	SD: n = 6	Fasted at least 10 h	SD: Same as CTR20140854	Rich SD: 108
			MD, QD × 7 days, 9A + 3P 400 mg	MD: n = 9		MD: Same as CTR20150048	MD: 279
CTR20150123	Healthy volunteers	R, 2 × 2 cross-over	100 mg	n = 14 or 15^b^	Fasted at least 10 h or high-fat meal	Pre-dose and post-dose 0.5, 1, 2, 3, 4, 5, 6, 8, 10, 12, 24, 36, 48, 72 and 96 h	Rich: 464
Phase 1b							
CTR20150549	Patients	R, DB, PC, PL, MAD	QD × 7 days, 1:1:1:1, placebo, 30, 100, 200 mg	n = 18	Fasted at least 4 h	Day 1 pre-dose and post-dose 0.75, 2, 3, 4, 5, 8, 12, 24 h; day 5, 6, 7 pre-dose; day 7 post-dose 0.75, 2, 3, 4, 5, 8, 12, 24, 36, 48, 72 and 96 h	Rich: 432
Phase 2							
CTR20170624 (NCT03458481)	Patients	R, OL, PL, MD	QD × 12 weeks, 1:1	Sparse: n = 107	Sparse: Fasted (at least 2 h before or after a meal)	Sparse: Week 1, 2, 4, 6, 8, 10, 12 pre-dose	Sparse: 872
			100 mg/sofosbuvir400 mg, 200 mg/sofosbuvir 400 mg	Rich: n = 22	Rich: Fasted at least 10 h	Rich: Day 1 and week 2 or 4 pre-dose and post-dose 1, 2, 3, 4, 6, 8, 10, 12 and 24 h	Rich: 426

R, Randomized; DB, Double blind; PC, Placebo control; PL, Parallel; SAD, Single ascending dose; MAD, Multiple ascending dose; OL, Open label; SD, Single dose; MD, Multiple dose; QD, Once daily; A, Active drug; P, Placebo.

Rich and sparse mean rich and sparse sampling schedule, respectively. Rich sampling entailed serial blood sapling at defined time points, and sparse sampling (single sample) entailed blood collection at all study visits.

^a^ No. of subjects who administered active drug yimitasvir.

^b^ 14 subjects completed the clinical trial under fasted condition, while 15 subjects completed the clinical trial under fed condition (high-fat meal).

All studies were conducted in accordance with the Declaration of Helsinki. Study protocols were approved by local ethics committees. Written informed consent was obtained from all subjects prior to study.

### Bioanalytical Methods and Data Handling

A fully validated liquid chromatography-tandem mass spectrometry (LC-MS/MS) method determining yimitasvir concentration in phase 1 and phase 1b studies had been reported elsewhere (Zhang et al., 2018; Zhao et al., 2019).

Plasma concentration of yimitasvir in phase 2 study was analyzed using a validated LC-MS/MS method equipped with a Shimadzu LC-30AD liquid chromatography-SCIEX API6500 mass spectrometer. Chromatographic retention and separation were achieved on a XBridge Peptide BEH C18, 50 × 2.1 mm, 3.5 μm column. Gradient elution was used with 2 mM ammonium acetate in water with 0.1% formic acid as mobile phase A and acetonitrile:methanol (30:70, v:v) as mobile phase B at a flow rate of 0.5 ml min^−1^. The column temperature was maintained at 45°C. Quantitation was accomplished in positive mode with precursor-to-production pairs of m/z 428.5→315.3 for yimitasvir and m/z 432.5→319.5 for the internal standard d8-DAG (isotope-labelled yimitasvir), respectively.

The calibration curve was linear in the range of 5.00–5000 ng·l^−1^ for both methods, and the lower limit of quantification (LLOQ) was 5.00 ng·l^−1^. Accuracy and precision were within the acceptable criteria of ± 15% for quality control (QC) samples and of ± 20% for LLOQs. Both methods were fully validated in accordance with National Medical Products Administration (NMPA) of China, U.S. Food and Drug Administration (U.S. FDA) and European Medicines Agency (EMA) guidelines. These two bioanalytical methods were not cross-validated.

Pharmacokinetic data with an absolute value of conditional weighted residual (CWRES) |CWRES| > 6 in the structural models were regarded as outliers. Outliers were omitted because these observations had a potential to negatively influence the convergence and/or poor estimation precision of parameters. If outliers were removed in the process of model development, the final model was re-run with or without the outliers to assess the potential influence on parameter estimates. If the frequency of LLOQ data was less than 10%, PK samples below LLOQ were excluded from model development. Otherwise, Beal’s M3 method was used for handling the LLOQ data (Beal, 2001). For covariates with missing values in less than 10% of subjects, continuous covariates were imputed as the population median, while a new category of ‘missing’ is generated for categorical covariates. No formal covariate screen procedure would be conducted if the covariates missed in more than 10% of the subjects.

### Model Development

Population pharmacokinetic analysis of yimitasvir was performed by Phoenix NLME software (Version 8.1, Certara) using the first-order conditional estimation-extended least squares (FOCE-ELS) method.

The structural model was first tested by fitting a one-compartment or two-compartment model to the log-transformed PK data. Different absorption models including first-order absorption with or without a lag time, sequential zero-first order absorption, transit absorption and saturable Michaelis-Menten absorption model, were also tested. The optimal structural model was selected based on the Akaike Information Criteria (AIC), minimization success, visual inspection of goodness-of-fit plots and individual fit.

Inter-subject variability was estimated by exponential model for PK parameters as follows (Eq. 1):

$$\theta_{\text{i}}=\theta_{\text{TV}}{\text{×e}}^{\eta \text{i}},$$ (1)

where θ_i_ is the parameter estimation for the *i*th individual, θ_TV_ is the typical value of the parameter estimation in the population, η_i_ is a random variable which assumed to be normally distributed with a mean of 0 and a variance of ω^2^. Proportional error model and proportional plus additive error model were tested as residual error models.

Following the development of structural model, the dose effect on bioavailability was evaluated first due to the less than dose-proportional profile of yimitasvir. Sigmoidal maximum effect (E_max_) (Eq. 2) and linear models (Eq. 3) were tested to quantify the relationship between bioavailability and dose:

$$\{\begin{array}{c} F={\text{θ}}_{\text{F }},\text{Dose}\leq 100\;\text{mg}\\ F={\text{θ}}_{\text{F}}-{\text{F}}_{\text{max}}\times \frac{\left(\text{Dose}-100\right)}{\left({\text{F}}_{50}+\left(\text{Dose}-100\right)\right)},\text{Dose}>100\;\text{mg} \end{array},$$ (2)

where θ_F_ is the bioavailability in individuals who received ≤100 mg yimitasvir, which was fixed to 1. F_max_ is the maximal reduction in bioavailability and F_50_ is the dose associated with a half-maximal reduction in bioavailability.

$$\{\begin{array}{c} F={\text{θ}}_{\text{F }},\text{Dose}\leq 100\;\text{mg}\\ F={\text{θ}}_{\text{F }}-\text{Alpha}\times \frac{\left(\text{Dose}-100\right)}{100},\text{Dose}>100\;\text{mg} \end{array},$$ (3)

where Alpha is a slope term determining the relative change in bioavailability for each 100 mg increase in yimitasvir dose.

Subsequently, different covariates were tested using a stepwise forward inclusion [a decrease in objective function value (OFV) of > 6.63, *p* < 0.01] and a stricter backward exclusion procedure (an increase in OFV of > 10.83, *p* < 0.001). The covariates included age, gender, body weight (BW), body mass index (BMI), baseline haemoglobin (HGB), baseline aspartate aminotransferase (AST), baseline alanine aminotransferase (ALT), baseline albumin (ALB), baseline total bilirubin (TBIL), baseline creatinine clearance (CLcr) calculated by Cockcroft-Gault formula (Cockcroft and Gault, 1976), co-medication of sofosbuvir, disease status (healthy volunteers *vs* patients) and food effect.

The effect of continuous covariates was modeled using a power function after normalization by the population median (Eq. 4):

$$\theta_{\text{i}}\;=\;\theta_{\text{TV}}\text{×}{\left({\text{cov}}_{i}/{\text{cov}}_{\text{median}}\right)}^{\theta \text{x}}.$$ (4)

The effect of categorical covariates was modeled using exponential format as follows (Eq. 5):

$$\theta_{\text{i}}=\theta_{\text{TV}}{\text{×e}}^{\theta x\mathrm{cov}}=\text{ k,}$$ (5)

where cov_i_ and cov_median_ represent covariate values for the *i*th individual and the population median, respectively. k is a categorical variable, and θ_x_ is a coefficient used to describe the strength of the covariate effect.

When two covariates were highly correlated (r^2^ > 0.7) such as ALT *vs* AST, only the most significant one was reserved in the model if both covariates were considered to be significant for the same PK parameter during univariate screen process.

### Model Evaluation and Simulation

The predictive performance of the final model was assessed by prediction-corrected visual predictive check (pcVPC) method using 1,000 trial replicates stratified by study. The observed data were plotted against the median, the 5th and 95th percentiles of predicted concentrations. The model was considered to be precise if the observed data were evenly distributed around the median prediction and within the 90% predicted intervals. In addition, a bootstrap procedure (n = 1,000) sampling with replacement from the original data was used to further test the robustness of the final model.

Individual empirical Bayes estimates PK parameters from the final model were used to predict the steady state exposure of yimitasvir. The simulated dosing regimen was yimitasvir 100 mg once daily for 12 consecutive weeks. The sensitive plot was plotted to present the effect of a significant covariate on yimitasvir exposure [steady state area under curve (AUC_ss_), steady state minimum concentration (C_trough,ss_) and steady state maximum concentration (C_max,ss_)]. The steady state exposure was calculated using PK parameters with incorporation of the isolated effect from the covariate and with other unaffected PK parameters fixed to the typical value. The overall exposure variability of the population was compared with the variability from those significant covariates.

## Results

### Population Characteristics

A total of 3,540 pharmacokinetic records (99.1%) from 219 subjects (72 healthy volunteers and 147 HCV infection patients) were included for model development. 31 (0.9%) samples below LLOQ were excluded from analysis. All CWRES ranged between −6 and 6. No PK data points were identified as outliers in the structural model and were excluded in the process of model development. Baseline demographics and subject characteristics were summarized in Table 2.

**TABLE 2:** Baseline demographics.

Continuous covariates^a^	Patients (n = 147)	Healthy volunteers (n = 72)	Total (n = 219)
Age (years)	46 ± 12 (47, 23–75)	27 ± 5 (27, 18–38)	40 ± 13 (38, 18–75)
BW (kg)	64.4 ± 12.2 (62, 44–100)	62.4 ± 6.0 (61.5, 51–77)	63.7 ± 10.6 (62, 44–100)
BMI (kg m^−2^)	23.8 ± 3.0 (23.7,18.0–31.6)	22.2 ± 1.6 (22.2, 19.0–25.0)	23.3 ± 2.8 (23.0, 18.0–31.6)
HGB (g l^−1^)	147 ± 14.7 (146, 111–183)	148 ± 13.5 (151, 114–171)	147 ± 14.3 (148, 111–183)
ALB (g l^−1^)	44.9 ± 3.5 (44.6, 36.9–54.3)	47.0 ± 2.4 (47.1, 41.4–52.4)	45.6 ± 3.3 (45.9, 36.9–54.3)
ALT (IU l^−1^)	72.3 ± 75.4 (52, 10.3–666)	14.4 ± 6.4 (13, 5.0–34)	53.3 ± 67.5 (31.6, 5.0–666)
AST (IU l^−1^)	51.0 ± 39.2 (41.1, 19.2–301)	17.2 ± 4.1 (16.0, 10.0–33.0)	39.9 ± 35.9 (30.1, 10.0–301)
TBIL (mg dl^−1^)	15.1 ± 6.2 (13.9, 3.1–37.7)	11.6 ± 4.1 (11.0, 3.7–21.4)	14.0 ± 5.8 (12.9, 3.1–37.7)
CLcr (ml min^−1^)^b^	109 ± 30.4 (103, 48.0–227)	97.6 ± 11.6 (95.5, 77.6–128)	105 ± 26.3 (99.8, 48.0–227)
Categorical covariates^a^			
Gender			
Male	72 (49.0)	54 (75.0)	126 (57.5)
Female	75 (51.0)	18 (25.0)	93 (42.5)
Co-medication sofosbuvir			
*Yes*	129 (87.8)	0 (0)	129 (58.9)
*No*	18 (12.2)	72 (100)	90 (41.1)
Food status			
0 (Fasted at least 10 h)	129 (87.8)	71 (98.6)^c^	200 (91.3)^c^
1 (Fasted at least 4 h)	18 (12.2)	0^c^	18 (8.2)^c^
2 (High-fat meal)	0 (0)	15 (20.8)^c^	15 (6.8)^c^

SD, standard deviation; BW, body weight; BMI, body mass index; HGB, hemoglobin; AST, aspartate aminotransferase; ALT, alanine aminotransferase; ALB, albumin; TBIL, total bilirubin; CLcr, creatinine clearance.

^a^ Mean ± SD (median, range) for continuous covariates and n (%) for categorical covariates.

^b^ Creatinine clearance (CLcr) was calculated by Cockcroft-Gault formula: CLcr (ml min^−1^) = [140 – Age (year)] × BW (kg) × 0.85 (if female)/[72 × Cr (mg dl^−1^)].

^c^ The total percent was not 100% because there are 14 identical subjects both in the group of food = 0 and in the group of food = 2

### Model Development

**FIGURE 2:**
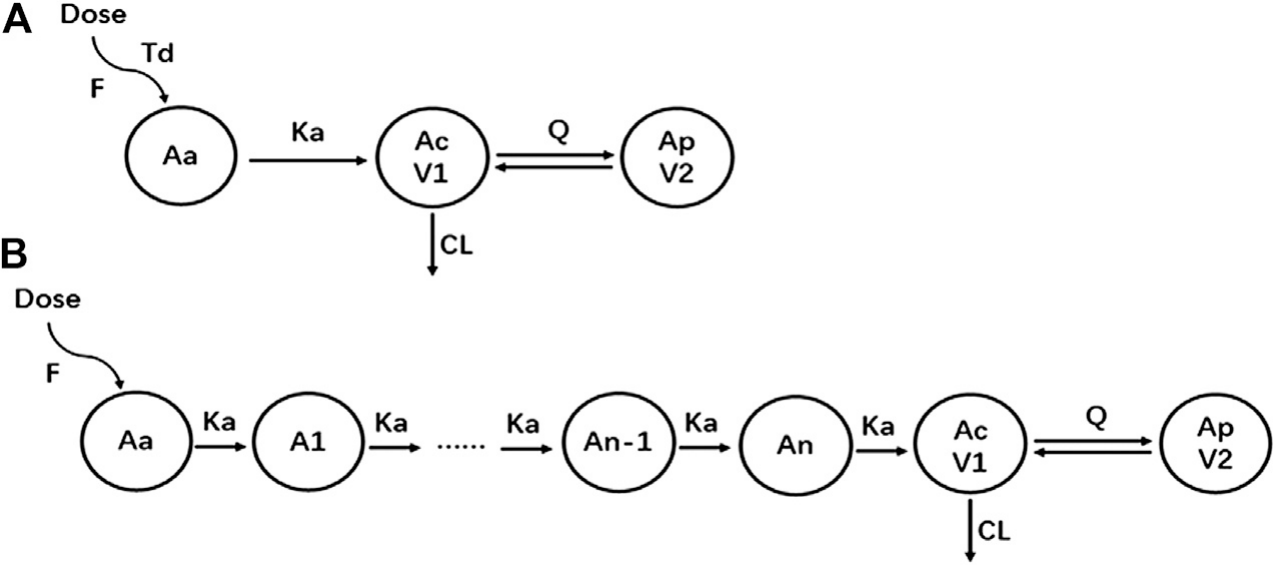
The structure of pharmacokinetic models. **(A)** Sequential zero-first order absorption model; **(B)** Transit absorption model.

A two-compartment model with sequential zero-first order absorption and first-order elimination was investigated to describe the pharmacokinetics of yimitasvir. The structural model was first established using intensive PK data from single ascending dose and multiple ascending dose (CTR20140854, CTR20150048 and CTR20170932) in healthy volunteers. A two-compartment model with first-order elimination was superior to a one-compartment model to describe yimitasvir PK profile. For the tested absorption model including first-order absorption with or without a lag time, sequential zero-first order absorption (Tong et al., 2018a; Tong et al., 2018b), transit absorption (Jain et al., 2011; Hu et al., 2018) and saturable Michaelis-Menten absorption (Wu et al., 2012), transit absorption model had the smallest AIC value and fitted the data best from the goodness-of-fit plots. Sequential zero-first order absorption model also performed well. When the full PK data were used, transit absorption model converged difficultly and slowly. Finally, we chose sequential zero-first order absorption model. Residual variability was modeled by proportional error model. The structure of sequential zero-first order and transit absorption model was plotted as Figure 2.

After the base structural model was established, the dose effect on PK parameters was tested first. The linear model was simple and adequate to describe the effect of dose on bioavailability. With incorporation of linear model, the objective function value (OFV) decreased by 82 units. Model fitting was improved significantly especially for high concentration data points. The typical value of Alpha was fixed to 0.129 according to the model result using data from SAD and MAD studies, which indicated that the bioavailability of yimitasvir decreased 12.9% for each 100 mg dose increase.

Subsequently, a stepwise forward inclusion and a backward exclusion procedure were performed to screen different covariates. During univariate screen procedure, covariates of ALT and AST, which were highly correlated (r^2^ = 0.93), were statistically significant on the same PK parameter of apparent clearance. ALT was considered to be more significant (decrease in OFV 18.291) than AST (decrease in OFV 12.675). As a result, the effect of ALT on clearance was reserved for further covariate screening. Following the stepwise forward inclusion process, food status was identified to be a significant covariate on first-order absorption rate (Ka) and bioavailability (F), gender and ALT were significant covariates on apparent clearance and disease status on duration of zero-order absorption (Td). None of these significant covariates was removed during the process of backward elimination. The final model parameter estimates and their associated precisions (percent confidence of variation, CV%), including the effect of significant covariates (*p* < 0.001), were provided in Table 3. The typical values (for a healthy male volunteer with ALT value of 31.6 IU l^−1^ taking yimitasvir under fasted state) for apparent oral clearance (CL/F) and central volume of distribution (V1/F) were 13.8 l·h^−1^ and 188 l, respectively. Inter-individual variability for CL/F and V1/F and the covariance of them were 48.5%, 73.6% and 56.5%, respectively. The shrinkage values for CL/F and V1/F were 3.25% and 8.69%, respectively, which indicated that individual empirical Bayes estimates PK parameters could be used to predict yimitasvir exposures (Savic and Karlsson, 2009). In addition, the ε-shrinkage was 4.83%. High-fat meal decreased Ka and F by 90.9% and 38.5%, respectively. Male subjects had a 22.2% higher yimitasvir CL/F than females. Baseline ALT was another significant covariate on apparent clearance. Duration of Zero-order absorption duration was longer in healthy volunteers (2.17 h) than that in patients (1.43 h). All the parameters were estimated with good precisions.

**TABLE 3:** Parameter estimates of the final population pharmacokinetic model.

Parameter	Estimate (RSE%)	95% CI	Bootstrap	
			Mean	95% CI
Fixed effect				
Ka (h^−1^)	1.31 (7.2)	1.13–1.49	1.38 (13.7)	1.09–1.80
CL/F (l h^−1^)	13.8 (4.1)	12.7–14.9	13.8 (3.7)	12.8–14.8
V1/F (l)	188 (7.1)	162–215	189 (5.6)	167–209
Q/F (l h^−1^)	3.96 (14.7)	2.82–5.10	4.04 (16.8)	2.92–5.53
V2/F (l)	58.6 (6.9)	50.7–66.4	59.1 (9.1)	50.0–70.9
Td (h)	2.17 (4.4)	1.98–2.36	2.22 (7.2)	1.97–2.56
Alpha	0.129 (fixed)	—	—	—
F	1 (fixed)	—	—	—
Ka ∼ Food1	−1.71 (−5.5)	−1.89∼−1.52	−1.72 (−10.5)	−2.09∼−1.35
Ka ∼ Food2	−2.40 (−3.7)	−2.58∼−2.23	−2.43 (−7.4)	−2.79∼−2.08
CL/F ∼ Female	−0.251 (−14.2)	−0.322∼−0.181	−0.250 (−14.6)	−0.326∼−0.177
CL/F ∼ ALT	−0.0950 (−21.2)	−0.135∼−0.0554	−0.0955 (−15.3)	−0.127∼−0.0647
F ∼ Food1	−0.341 (−26.3)	−0.517∼−0.166	−0.312 (−34.9)	−0.523∼−0.0904
F ∼ Food2	−0.485 (−31.2)	−0.782∼−0.189	−0.473 (−19.8)	−0.644∼−0.263
Td ∼ Patient	−0.416 (−10.7)	−0.504∼−0.329	−0.403 (−27.9)	−0.634∼−0.192
Random effect				
CL/F	0.485 (3.8)	0.449–0.521	0.483 (5.8)	0.427∼0.538
V1/F	0.736 (6.9)	0.637–0.834	0.729 (11.0)	0.571∼0.886
CL/F ∼ V1/F	0.565 (5.1)	0.509–0.622	0.562 (8.1)	0.473∼0.651
Residual error				
σ	0.305 (0.7)	0.300∼0.309	0.304 (3.4)	0.284∼0.325

### Model Evaluation and Simulation

**FIGURE 3:**
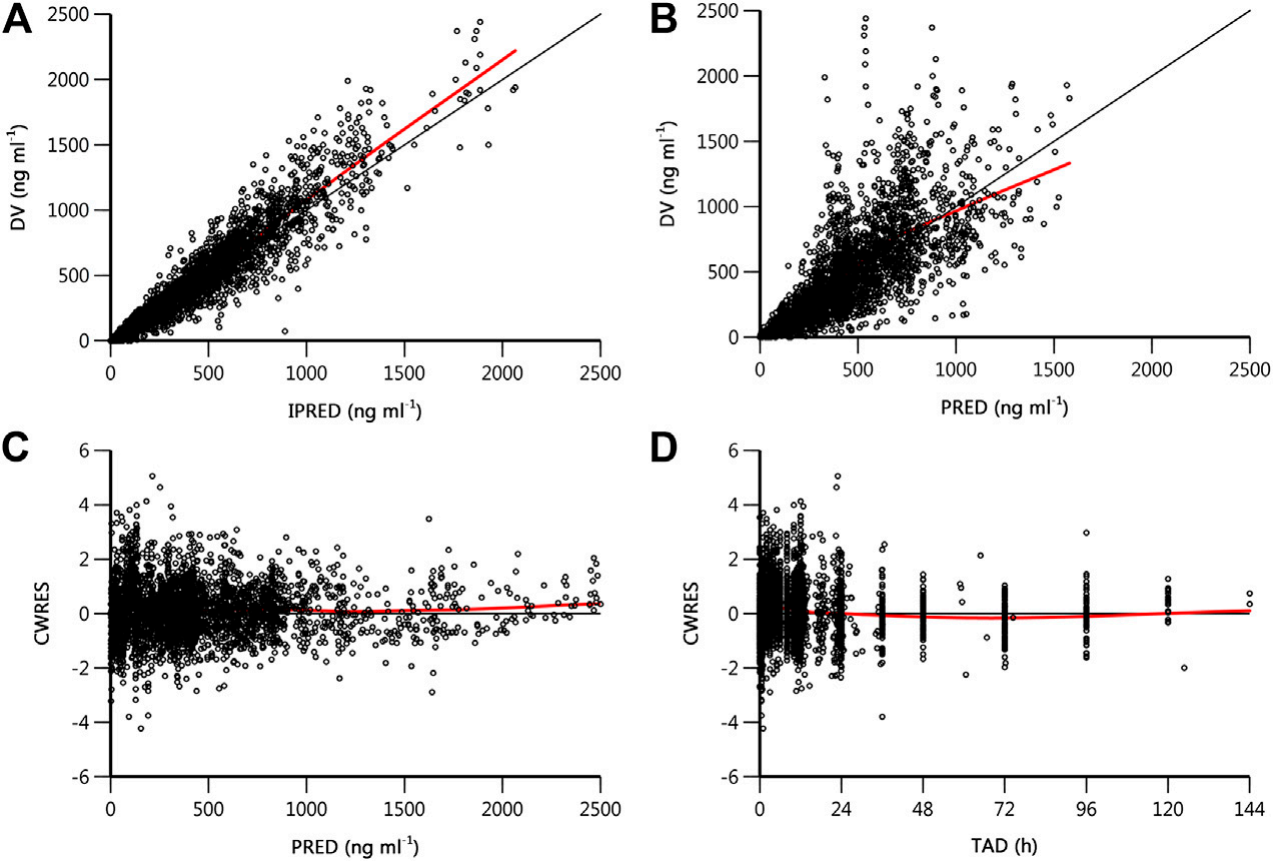
Goodness-of-fit plots of final population pharmacokinetics model. **(A)** Observed drug concentration (DV) *vs*. individual prediction (IPRED); **(B)** DV *vs*. population prediction (PRED); **(C)** conditional weighted residual (CWRES) *vs*. PRED; **(D)** CWRES *vs*. time after dose (TAD). The black line is the line of unity or the zero reference line, and the red line is the result of locally weighted scatterplot smoothing (loess).

Goodness-of-fit plots for the final pharmacokinetic model are shown in Figure 3. The population and individual predictions agreed with the yimitasvir observations well, but there was a trend of under-prediction at high concentration data points (Figures 3A,B). Most of these data were from 200 mg group of phase 2 study. One possible assumption for the bias was the lack of precise dosing history in phase 2 trial although the last dosing time before each PK sampling was recorded. Most CWRES ranged from y = ±4 and were evenly distributed at y = 0 (Figures 3C,D) with no obvious bias over the population predictions and time indicating the proper choice of proportional model for the residual variability.

**FIGURE 4:**
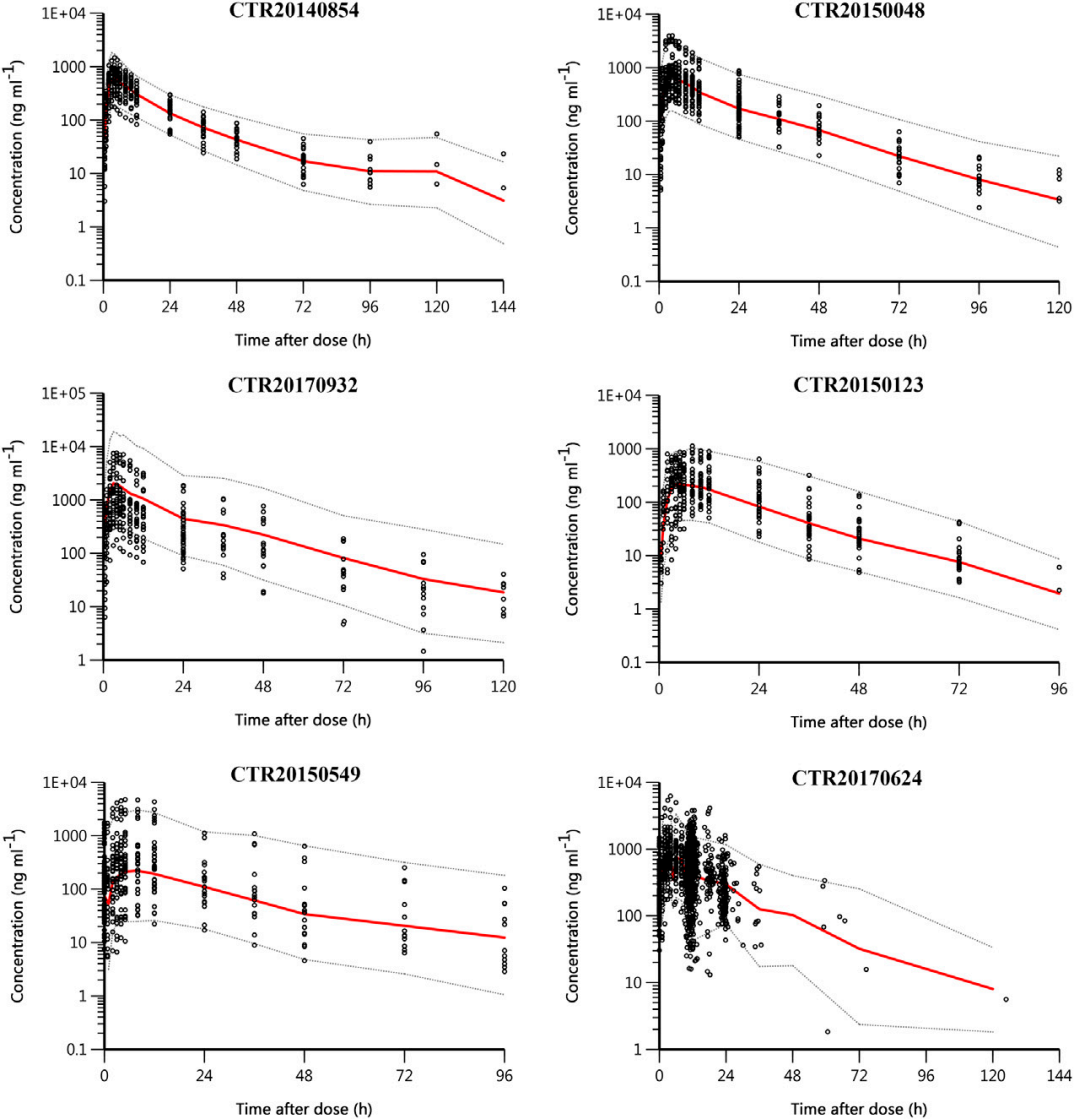
Prediction-corrected visual predictive check (pcVPC) plot stratified by study. Black circles are observed yimitasvir concentrations, red solid lines represent the median of predicted concentrations, and gray dashed lines represent the 5th and 95th percentiles of predicted concentrations.

Figure 4 shows the pcVPC plot stratified by study. Apart from study CRT20170932, the observed data were evenly distributed around the median prediction and were mostly within the 90% percentiles of the predicted concentrations in other studies. Study CRT20170932 was a supplementary clinical trial of studies CTR20140854 and CTR20150048. The objective of this study was to evaluate the safety and pharmacokinetics of yimitasvir when administered at higher doses, namely 600 mg single dose and 400 mg once daily for consecutive 7 days. According to the result of the final model, the relative bioavailability of 200, 400 and 600–100 mg was 87.1, 61.3 and 45.5%, respectively. The relatively bioavailability of 400 and 600 mg was somewhat over-predicted, while the relative bioavailability of 200 mg was appropriate. Hence, except that study CRT20170932 was over-predicted, the pcVPC plots across the rest of the studies seemed reasonably well.

In the bootstrap for the final model, all the 1,000 replications ran successfully. The population parameter estimates were close to the median values from bootstrapping analysis and fell within 95% confidence intervals (Table 3), suggesting that the final model was robust and accurate.

**FIGURE 5:**
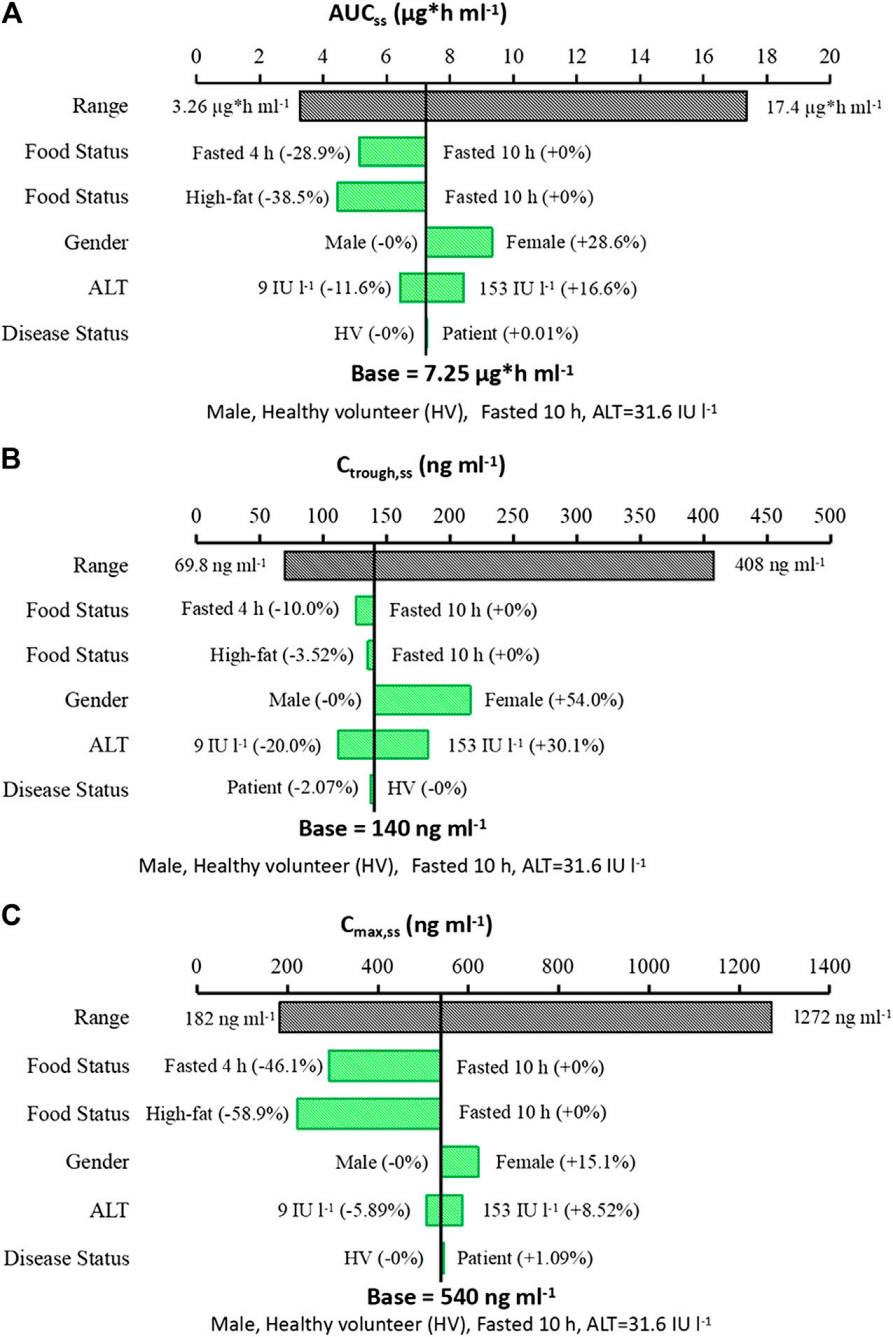
Sensitive plot comparing the effect of covariates on yimitasvir steady state exposure. **(A)** AUC_ss_; **(B)** C_trough,ss_; **(C)** C_max,ss_. A typical subject is a healthy male volunteer with ALT value of 31.6 IU l^−1^ taking yimitasvir under fasted state more than 10 h. The black bar represents the 5th to 95th percentile of the exposure calculated using empirical Bayes estimates of the population after administration of yimitasvir 100 mg once daily for 12 consecutive weeks. Continuous covariates were evaluated at the 5th to 95th percentile of the population.

The influence of significant covariates on predicted steady state exposure (AUC_ss_, C_trough,ss_ and C_max,ss_) was presented in Figure 5. The result revealed that food status had a great impact on yimtasvir AUC_ss_ and C_max,ss_, but not C_trough,ss_. AUC_ss_ and C_max,ss_ decreased by 38.5% and 58.9% after a high-fat meal, respectively. The C_trough,ss_ of females was 54% higher than that of males, while AUC_ss_ and C_max,ss_ of females were less than 30% higher than that of males. The magnitude of ALT on the steady-state exposure of yimitasvir was mild (∼30%) for healthy volunteers with extreme ALT values (5th and 95th percentiles) relative to the typical healthy volunteers. There was no difference in exposures between patients and healthy volunteers.

## Discussion

This population PK analysis was established using rich and sparse PK samples collected from six clinical trials including 72 healthy volunteers and 147 HCV infection patients. A two-compartment model with a sequential zero-first order absorption and first-order elimination could described the PK profiles of yimitasvir well. After a stepwise forward inclusion and backward exclusion covariate screen procedure, statistically significant covariates in the final model were food status on Ka and F, gender and ALT on CL/F, and disease status on Td.

Results from phase 1 SAD study in healthy volunteers suggested that the exposure (AUC and C_max_) of yimitasvir increased less than proportionally in the range of 30–600 mg. Similar exposures were found between 400 and 600 mg groups indicating a limited absorption. Less than dose proportionality was also observed in phase 2 study in patients after oral administrations of 100 or 200 mg yimitasvir in combination with 400 mg sofosbuvir. Sofosbuvir did not affect the pharmacokinetic properties of yimitasvir, which had been proven by other drugs targeting HCV NS5A protein like velpatasvir (Brieva et al., 2017) and ledipasvir (German et al., 2016). However, the exposure of yimitasvir was dose-proportional in phase 1b trial in patients with chronic HCV genotype 1 infection after single and multiple oral dosing from 30 to 200 mg. The mechanism behind the difference in the PK linearity of yimitasvir between phase 1b and the other trials is unclear. One significant difference in clinical trial design was that subjects in phase 1b trial administered yimitasvir in the evening after dinner for more than 4 h, while subjects in other trials took the drug in the morning under fasted state. The inefficiency of gastric emptying resulted in decrease of absorption rate with a median time to peak concentration (T_max_) of 4–12 h and a 40% AUC decrease when compared to phase 1 SAD trial. The decrease in exposure may contribute to the linearity of yimitasvir in phase 1b trial.

Food status was a significant covariate affecting the absorption rate (Ka) and bioavailability (F). In this population PK analysis, food was classified into three categories. Subjects who took the drug under fasted and fed (high-fat meal) condition were classified as food = 0 (as reference) and food = 2, respectively, while subjects in phase 1b trial who administered yimitasvir in the evening after dinner more than 4 h were classified as food = 1. Administration with a high-fat meal or in the evening after dinner more than 4 h resulted in a 38.5% and a 28.9% decrease in AUC_ss_, a 3.52% and 10.0% decrease in C_trough,ss_, and a 58.9% and 46.1% decrease in C_max,ss_, respectively (Figure 5).

According to the food effect study results, a high-fat meal resulted in approximate 50% and 63% decrease in yimitasvir AUC and C_max_, respectively. While the model simulated that administration with a high-fat meal, AUC_ss_ and C_max,ss_ decreased about 38.5% and 58.9%, respectively. The impact of food status on yimitasvir PK was somewhat under-estimated. One reasonable explanation was that the food status for all phase 2 patients was treated as 0 (fasted more than 10 h) for convenience. However, the food status in patients of phase 2 was different. For subjects with intensive sampling on Day 1 and Week 2 or 4, they were fasted more than 10 h before they took yimitasvir. While for subjects with sparse sampling, the protocol required that the drug was taken at least 2 h before or after a meal (Table 2). There were no exact records for these subjects about the time interval between administration of yimitasvir and meals. It could be speculated that the exposure under fasted state (food = 0) was lower than that in the real situation, which leaded to an under-estimation of the food impact on yimitasvir PK.

The decrease in steady state exposures after a high-fat meal might be due to the solubility of yimitasvir. Yimitasvir exhibited a pH-dependent solubility profile. In the fasted condition, yimitasvir solubility increased due to a low gastric pH in the stomach, while a high-fat meal resulted in higher gastric pH (Carver et al., 1999). It could be speculated that the impact of 4 h fasted on yimitasvir exposure was lower than the impact of 2 h fasted. In phase 2 study, yimitasvir was taken at least 2 h before or after a meal in most patients and the primary endpoint of SVR12 rates were achieved 100%. It seemed that taking yimitasvir at least 2 h before or after a meal had little impact on yimitasvir response. Hence, it was recommended that yimitasvir was administered at least 2 h before or after a meal instead of at least 4 h.

Gender was found to be a significant covariate for yimitasvir CL/F. Female subjects had lower apparent clearance than male subjects. The steady state exposures (AUC_ss_, C_trough,ss_, C_max,ss_) in female subjects were 15.1–54.0% higher than male subjects. The phenomenon of lower CL/F values in females was also found in other NS5A inhibitors such as daclatasvir (Chan et al., 2017), velpatasvir (U.S. Food and Drug Administration, 2016) and ledipasvir (U.S. Food and Drug Administration, 2014). However, the underlying mechanism was not clear. In our analysis, body weight was not a significant covariate on yimitasvir PK, so the increase of exposure in female subjects could not be explained by the lower body weight in female subjects. Another significant covariate on CL/F was baseline ALT. The higher the baseline ALT levels, the lower the CL/F values, the higher AUC_ss_ would be. However, in our dataset, females tended to have lower baseline ALT values than males both in healthy volunteers (female: 11.8 ± 5.33 IU l^−1^, n = 18, mean ± SD; male: 15.3 ± 6.60 IU l^−1^, n = 54) and in patients (female: 60.8 ± 85.8 IU l^−1^, n = 75; male: 84.4 ± 61.0 IU l^−1^, n = 72). Baseline ALT values also could not explain the higher exposures in females.

Literatures had reported that the serum ALT levels decreased to normal after anti-virus treatment in HCV patients (Kawamura et al., 2005). A population pharmacokinetic analysis of daclatasvir (a NS5A inhibitor) studied only in patient population demonstrated that time-varying ALT was a significant covariate on CL/F (Chan et al., 2017). In our analysis, we just evaluated the effect of baseline ALT level on yimitasvir PK, not the time-varying ALT. One reason was that time-varying ALT was not identified as a significant covariate on the PK of some NS5A inhibitor drugs, such as velpatasvir (U.S. Food and Drug Administration, 2016) and ledipasvir (U.S. Food and Drug Administration, 2014), when both healthy volunteers and HCV patients were included in the population pharmacokinetic analysis. It was more meaningful to study the effect of time-varying ALT on yimitasvir PK when the population included only patients. In addition, although time-varying ALT had a significant impact on daclatasvir clearance, the effect on AUC_ss_ was small. Hence, the time-varying ALT was not evaluated in our analysis finally.

In phase 2 clinical trial, yimitasvir 100 or 200 mg was administered once daily for consecutive 12 weeks in combination with 400 mg sofosbuvir in HCV infection patients. The primary endpoint of SVR12 rates were achieved 100% in both 100 mg yimitasvir/400 mg sofobuvir and 200 mg yimitasvir/400 mg sofobuvir groups. The adverse reaction rates were comparable between 100 mg (35.9%) and 200 mg (36.9%) groups. The most common adverse reactions were neutropenia (3.9%), leukopenia (3.1%), hypercholesterolemia (3.1%) and fatigue (3.1%). All of these adverse reactions were grade 1 or 2 in severity. No dose-response relationship for efficacy and safety was identified in phase 2 clinical trial. Considering the high response rate and favorable safety profiles of yimitasvir in phase 2 clinical study, the significant covariates of gender and baseline ALT on clearance seemed not clinically relevant. Except for gender, other baseline demographics such as age and BW had no effect on yimitasvir PK properties. It seemed that there was no need to adjust dosage based on these factors. But due to the small sample size of phase 2 study, this conclusion should be further validated in a larger patient population.

There was no difference in PK parameters between patients and healthy volunteers, except for parameter Td, which was a little longer in healthy volunteers than that in patients. The difference in Td between the two kinds of population did not result in exposure differences.

This is the first time to establish a population PK model of a new HCV NS5A inhibitor, yimitasvir, in Chinese healthy volunteers and patients with chronic HCV genotype 1 Infection. It is very helpful for us to know statistically significant covariates affecting the pharmacokinetic property of yimitasvir. This work can be used as a basis for subsequent population PK model development including more patient data from phase 3 trial.

One limitation of our analysis was the relatively small sample size. There were only 147 HCV patients involved in the dataset, which made it inadequately to quantify the impact of disease related covariates. Another limitation was the population we studied were all Chinese. It was not clear that the ethnic differences in pharmacokinetics of yimitasvir between Chinese and other races.

In conclusion, the population pharmacokinetic model was demonstrated to be appropriate and effective to describe the pharmacokinetics of yimitasvir in Chinese population. Food status, disease status (healthy volunteers *vs* patients), gender and baseline ALT were identified as statistically significant covariates to affect yimitasvir pharmacokinetics. High-fat meal decreased absorption rate and bioavailability, so it is recommended to take yimitasvir at least 2 h before or after a meal. Considering the favorable safety profile of yimitasvir and 100% SVR12 rate in phase 2 study, the impact of gender and ALT on yimitasvir exposure seemed not to be clinically relevant. This conclusion should be further validated in a larger patient population from phase 3 clinical trial.

## Data Availability Statement

The original contributions presented in the study are included in the article/Supplementary Material, further inquiries can be directed to the corresponding authors.

## Ethics Statement

The studies involving human participants were reviewed and approved by local ethics committees. The patients/participants provided their written informed consent to participate in this study.

## Author Contributions

Y-jZ, H-mX, LL, and DW collected the data. X-dG analyzed the data and wrote the manuscript. X-dG, X-gT, RC and PH involved in the discussion of results. All the authors have read and approved the final manuscript.

## Funding

The work was supported by the “CAMS Innovation Fund for Medical Sciences” (CIFMS) (Grant No. 2016-I2M-1-010).

## Conflict of Interest

Y-jZ, H-mX, LL, and DW are employees of Sunshine Lake Pharma Co., Ltd.

The remaining authors declare that the research was conducted in the absence of any commercial or financial relationships that could be construed as a potential conflict of interest.
